# Effects of a transtheoretical model - based intervention and motivational interviewing on the management of depression in hospitalized patients with coronary heart disease: a randomized controlled trial

**DOI:** 10.1186/s12889-020-08568-x

**Published:** 2020-03-30

**Authors:** Xiaoyun Li, Silan Yang, Yishu Wang, Bingxiang Yang, Jingping Zhang

**Affiliations:** 1 Xiangya Nursing School of Central South University, 172 Tong Zi Po Road, Changsha, 410000 Hunan China; 2 Department of Nephrology, The Second Xiangya Hospital of Central South University, Changsha, Hunan China; 3 The Second Xiangya Hospital of Central South University, Changsha, Hunan China; 4 Health Sciences of Wuhan University, Wuhan, Hubei China

**Keywords:** Transtheoretical model, Motivational interviewing, Depression, Coronary heart disease

## Abstract

**Background:**

Depression is a major risk factor for the morbidity and mortality of cardiovascular disease. A transtheoretical model-based intervention and motivational interviewing have been used to change health risk behaviors and have demonstrated positive effects. To our knowledge, no studies of patients with coronary heart disease (CHD) have used a transtheoretical model-based intervention and motivational interviewing as an intervention to provide dynamic education. Therefore, this study aimed to determine the effects of the transtheoretical model-based intervention and motivational interviewing on the management of depression in hospitalized patients with CHD.

**Method:**

A randomized controlled trial was designed. A total of 110 participants were randomly divided into an intervention group (*n* = 55) and a control group (*n* = 55). The Hamilton Rating Scale for Depression and the Depression Prevention & Management Survey items (stages of change, perceived benefits, perceived barriers, process of change and self-efficacy) were used to collect data at all time points. Analysis of covariance, chi-square test and repeated measures analysis of variance were used to analyze the data.

**Results:**

After the intervention, there were more positive changes in stages of change, higher scores for the cognitive and behavioral levels, the perceived benefits, and self-efficacy, and lower perceived barriers and depression in the intervention group than in the control group. Finally, there were statistically significant differences in the depression scores at different time points in the intervention group (*F* = 17.814, *p* = 0.000 < 0.01).

**Conclusions:**

The study showed that a transtheoretical model-based intervention and motivational interviewing exert positive effects on the management of depression in hospitalized patients with CHD.

**Trial registration:**

Clinicaltrials.gov, NCT03953924 (Date assigned: 16/5/2019). Retrospectively registered.

## Background

In the twentieth century, coronary heart disease (CHD) was the most common cause of death in the United States [1]. The Chinese health service survey showed that approximately 100,000 ~ 320,000 people were suffering from CHD in mainland China in 2008 [2]. Studies have indicated that the global cardiovascular mortality rate is expected to increase by 50%, and the number of deaths from cardiovascular diseases in Asian countries will double by 2020 [3]. The high mortality and morbidity of CHD will aggravate the psychological burden of patients, such as inducing depression. A study reported that the prevalence of depression in patients with CHD ranged from 8.2 to 35.7% in men and from 10.3 to 62.5% in women [4]. The incidence of depression in the Chinese population was reported to be in the range of 4%~ 6% and as high as 14%~ 17% in patients with CHD [5].

Many studies have shown that the occurrence, development and prognosis of, as well as recovery from CHD are closely related to depression [6, 7]. Depression not only affects the patients’ state of an illness but also reduces their quality of life and their compliance to medication treatment regimens and lifestyle changes [8, 9]. In addition, depression is a major risk factor for the morbidity and mortality of cardiovascular disease [10, 11]. A study of 30,239 patients, with an average follow-up time of 5.95 years, found that patients with CHD who were depressed had a higher risk of death than those without depression [6]. Studies have also shown that depressive symptoms were associated with a higher probability of mental stress-induced myocardial ischemia in patients with CHD [12, 13]. Therefore, there is an urgent need to screen for and treat depression in patients with CHD.

The American Heart Association recommended screening for depression in patients with CHD and subsequently be given appropriate treatment [14]. At present, the treatment for depression in patients with CHD includes antidepressant administration [15]. However, these medications have side effects. Other methods of treatment are psychological interventions, which include psychological counseling, knowledge education, relaxation therapy [16], cognitive behavioral therapy and so on [17]. Depression has been alleviated after psychological therapies, but a systematic review in Cochrane suggested considerable uncertainty surrounding these effects [18]. In addition, the content of these psychological interventions and knowledge education were not based on dynamically changing knowledge or other needs, such as self-cognition or self-efficacy. These studies cannot provide an analysis of staged results in the process of psycho-behavioral change. From what was discussed above, it is imperative that a new and dynamic intervention is used to alleviate depression in patients with CHD.

The transtheoretical model (TTM) is a purposeful behavioral change model. It is a systematic study of people’s behavior change based on a variety of theories. The intervention based on TTM was designed to guide intervention methods to change dangerous behaviors [19]. TTM includes four parts: stages of change (SOC), process of change (POC), decisional balance (DB), and self-efficacy (SE). DB includes perceived benefits and perceived barriers. SOC is divided into five stages: precontemplation, contemplation, preparation, action, and maintenance [20]. Effective outcomes with TTM have also been observed with health risk behaviors such as smoking [21], unhealthy alcohol use [22] and weight control [23].

**Fig. 1:**
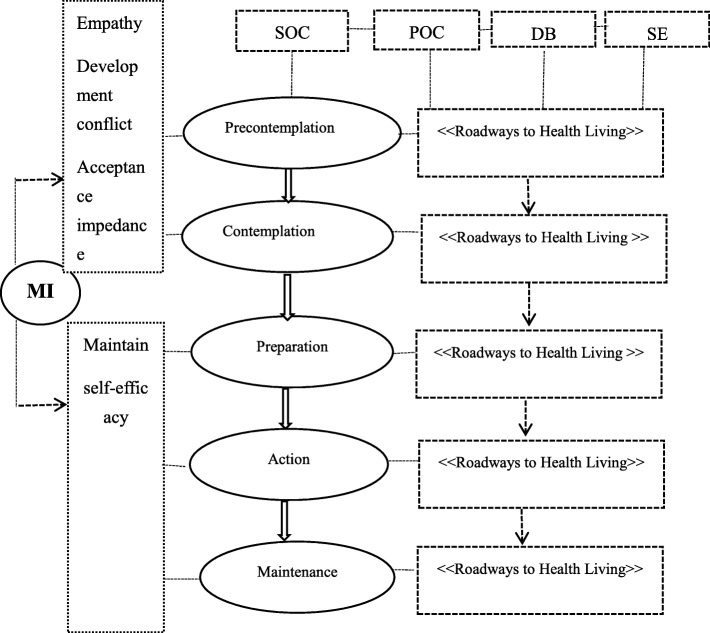
Conceptual framework

In addition, another method used to involve individuals in health behavior change is motivational interviewing (MI). MI takes an approach to interpersonal communication with patients as the center, which helps patients discover and overcome their ambivalence, thereby triggering behavioral changes [24]. It is an instructional way of communicating that invites people to think about their own situation and find their own solution [24]. A systematic review showed that MI might have favorable effects on changing patients’ depression and found that the effect of a single use of MI was better than that of cognitive-behavioral therapy [25]. Moreover, a study confirmed that educational and promotional interventions based on TTM and MI may change the behavior of psychiatric patients and positively influence their nutrition habits [26]. In summary, TTM-based interventions and MI have been used to change health behaviors and have demonstrated positive effects. To our knowledge, no studies using a TTM-based intervention and MI as an intervention to provide dynamic education have been conducted in patients with CHD. Therefore, we decided to determine the effects of a TTM-based intervention and MI on the management of depression in hospitalized patients with CHD and to provide patient-centered, accessible assistance to strengthen the patients’ ability to manage depression. Using an existing model as a foundation, we developed a mediating framework to depict the intervention pathway (Fig. 1).

## Methods

### Aim

The aim of this study was to determine the effects of a TTM-based intervention and MI on the management of depression in hospitalized patients with CHD. The study’s hypotheses were as follows: (1) The patients in the intervention group, compared with those in the control group, would show a positive shift in the stages of change and on scores for measures of cognition, behavior, perceived benefits, self-efficacy, and perceived barriers after the intervention; (2) The patients in the intervention group would achieve significant improvement in their depression compared with those in the control group.

### Trial design

The study was a single-blind, parallel, randomized, controlled trial and officially approved by the Institutional Review Board (IRB) of the Second Xiangya Hospital, Central South University (Changsha, Hunan Province, China) (NO. 2015S038). The patients were from three Central South University-affiliated general tertiary hospitals in Changsha, Hunan province, China. They were randomly divided into two groups by using a block randomization list with a block size of 4 at 1:1 by an investigator who did not participate in the study. A research assistant put the generated random numbers into opaque, consecutively numbered envelopes, sealed the envelopes, and then handed them to the enrolling researchers. When an eligible patient was recruited into the study, the enrolling researcher gave the patient a number and opened the sealed envelope with the same number. The enrolling researchers were blinded to the design of this study.

### Sample size

Considering the main purpose of the study, a 5% level of significance (two-tailed test) and a power of 0.90 were adopted: the sample size was calculated on the basis of the formula for sample size required to compare the mean of two samples [27]: ${n}_1={n}_2=2{\left[\frac{\left({u}_{\alpha }+{u}_{\beta}\right)}{\delta /\sigma}\right]}^2+\frac{1}{4}{u}_{\alpha}^2$; with reference to Yang’s study in patients with depression [28], effect size = δ/σ = (μ_1_ -μ_2_) / σ = (8.32–6.02)/3.34 = 0.689. In this study, μ_α/2_ = 1.960, β = 0.1, μ_0.01_ = 1.282, the sample size in each group: n_1_ = n_2_ = 2× ((1.960 + 1.282)/0.689)^2^ + 1/4 × 1.960^2^ ≈ 46; considering a 20 % attrition rate, 55 patients in each group would be adequate to meet parametric test assumptions.

### Recruitment and eligibility

The sample was 110 hospitalized patients with CHD in the Medicine-Cardiovascular Departments of three hospitals in Changsha. The eligibility criteria were as follows: (1) diagnosed with CHD (typical clinical angina manifestations, electrocardiographic changes, and coronary angiography) confirmed by the World Health Organization / International Cardiology guidelines from October 1997, with cardiac function graded from *I* to *Ш*; (2) had a level of education that was primary school or above; (3) volunteered to participate in the study; (4) had a clear consciousness and were able to express their wishes.

The exclusion criteria were as follows: (1) patients who had cerebrovascular accident, malignant oncology, malignant hypertension (systolic pressure > 180 and/or diastolic pressure > 100 mmHg); (2) patients who had psychiatric history or serious cognitive or consciousness obstacles; (3) patients who had participated in other similar research studies; (4) patients who could not be contacted by mobile phone or home phone.

### Consent procedures

Participants were informed of the purpose, significance, and methods of the research, the time that was required to fill out the questionnaire, and the possible risks and benefits of the study so that the patients could fully understand the content and how the study would progress.

### Interventions

#### Contact the hospital

The leader of research team contacted the hospital and obtained the consent of the relevant departments and cardiovascular internal medicine inpatient ward heads of the hospital to screen the potential research participants before the beginning of the study.

#### TTM-based intervention and MI

The patients in the intervention group received usual care (only in relation to their physical health) and the TTM-based intervention and MI, while those in the control group were given usual care. Trained nurses provided the patient interventions. The trained nurses consisted of one research leader and experienced nurses, all of whom had qualifications as national psychological counselors, who were familiar with and responsible for the CHD patients. The research leader informed the patients of the schedule for each session using the educational manual “Roadways to Health Living—guidelines for the management of depression”, which was developed by one psychologist, one experienced nurse, and related researchers. The experienced nurse was trained by the researcher about the intervention content and the process.

The MI was implemented 2 times, 20 min each time, and the trained nurses interviewed each patient face-to-face at bedside. The change stages of the patients’ behavior changes were identified by motivational interviewing. According to the change stage, the trained nurses adopted the face-to-face approach and incorporated corresponding measures to explain the contents of the manual named “Roadways to Health Living”. At the end of the baseline assessment, the patient was given the manual and guided reading. The TTM-based intervention was given 3 times in the form of a small course, 20 min each time, and each course was based on the manual.

The TTM-based intervention included 3 sessions: (1) In the first session, for patients who were in precontemplation or contemplation stage, we focused on the related knowledge of depression and CHD, in order to make patients understand the importance of management of depression to physical and mental health and the benefits of management of depression; (2) In the second session, for patients who were in reparation or action stage, we provided specific strategies and methods to help them manage depression and enhance their skills, We negotiated and made a action plan for change with patients; (3) In the third session, for patients who were in maintenance stage, we provide examples and experiences of successful improvement of depression, and affirm the efforts and achievements of patients to make them keep the will of action. Besides, we explored the situation that may hinder the action and provided the relevant coping strategies. Tables 1 and 2 show detailed descriptions of the intervention.

**Table 1:** Description of the interventions received by the patients in each group

Control group	Intervention group
Usual care: received support only in relation to their physical health.	Usual care MI^a^: Two sessions of 20-min face-to-face contact. (i) Risks, relevance, rewards, roadblocks, repetition; TTM^b^: Three sessions of 30-min face-to-face contact. (i) An introduction to depression and coronary heart disease; (ii) Strategies for management of depression; (iii) Experience in improving depression, situations that may interfere with action, and ways to deal with it. A manual about the management of depression covering all the above mentioned content. (i) TTM-based intervention+MI.

^a^*MI* motivational interviewing, ^b^*TTM* transtheoretical model

**Table 2:** Goals and strategies for the management of depression in each stage

Stage	Goal	Strategies	Techniques and processes of change
Precontemplation Contemplation	Finding and establishing the intrinsic motivation of behavior change through interviewing. Improve depression.	Provide knowledge about depression and CHD and the risks of depression (physical and psychosocial health). Encourage thinking about the benefits of improvements in depression.	Listening and guidance. Consciousness awakening, emotional arousal, self-reevaluation, environmental reevaluation and social liberation.
Preparation Action	Increase motivation and Confidence. Negotiate and make action plan for change. Take action to alleviate depression.	Provide specific strategies to prevent and manage depression. Help patients enhance their skills and develop action plans.	Timely confirmation. Awakening consciousness, helping relationships, self-emancipation, enhancing management and situational substitution.
Maintenance	Keep following up in hospital. Enhance patients’ self-confidence in managing depression.	Provide examples and experiences of successful improvement of depression, and affirm the efforts and achievements of patients. Explore situations that may hinder action. Provide appropriate coping strategies.	Helping relationships, self-emancipation, enhanced management, situational substitution, and stimulus control.

**Fig. 2:**
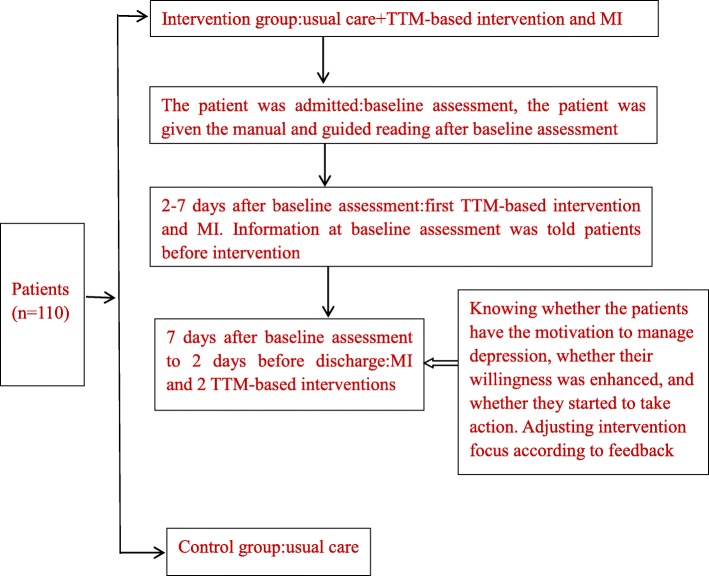
Intervention flow diagram

Sessions were held in a meeting room at 2 time points: 2–7 days after baseline assessment and 2 days before discharge in the two groups. The initial session included MI and the TTM-based intervention. The second session included MI and 2 TTM-based interventions. The intervention flow diagram is shown in Fig. 2.

All variables were collected at 2 time points: when the patient was admitted (T, obtain the baseline information) and 2 days before discharge (T0; i.e., the information obtained after the intervention) by another research assistant who was blinded to the study design and allocation of the participants used questionnaire. The data collection time between two times was approximately 2 weeks.

### Measures

#### Primary outcome measures

##### Hamilton rating scale for depression (HRSD)

Depression was measured by the HRSD. The scale was compiled in 1960 by Hamilton [29] and includes 24 items. A few items (4th, 5th, 6th, 12th, 14th, 16th, 17th, 18th, and 21st items) were evaluated with a 0–2-point scoring method, and the remaining items were evaluated with a 0–4-point scoring method. The higher the total scores were, the worse the depression. According to Davis JM [30], a score of 8 to 19 indicated no depressive symptoms, more than 20 indicated mild or moderate depression, and more than 35 indicated severe depression. In this study, Cronbach’s alpha was 0.819.

##### Depression Prevention & Management Survey (DPMS) items

SOC, DB, POC and SE were measured by DPMS. The original English edition of the questionnaire was compiled by the American Pro-Change Behavior Systems, lnc. Research Center. The form used in the present study was developed with authorization, preliminary work involving a pilot test, related literature review, modification based on a previous TTM intervention [28, 31], and consultation with six experts, including a psychologist, two experienced researchers in depression, a professor of cardiovascular internal medicine, and two bilingual nursing scholars with academic background. The index of content validity (CVI) was 0.988. The management of depression questionnaire mainly included four subscales: SOC, POC, DB, and SE subscale.

##### SOC subscale

The SOC subscale was used for measuring the stage of an individual’s depression management behavior and consisted of one item: “do you take effective ways to prevent and manage depression (enjoyable activities, keep trying to fight stress, and seek professional help if necessary and so on) in your daily life?”. There were five statements: precontemplation (‘no, and I’m not planing to do that in the next 6 months’), contemplation (‘no, but I plan to start in the next 6 months’), preparation (‘no, but I plan to start in the next 30 days’), action (‘yes, I have been doing that for more than 1 day and less than 6 months’), and maintenance (‘yes, I have been doing that for more than 6 months’). The patients were asked to select one suitable statement. The scale has good reliability (0.790) and retest reliability.

##### POC subscale

The POC subscale was used to evaluate individuals’ experiences or activities that can influence individuals to take effective measures to manage depression. It had 30 items and included cognitive and behavioral levels. Each item was scored from 1 (never) to 5 (always). The higher the score for a particular dimension was, the higher the frequency of use of that process. The Cronbach’s alpha of cognitive processes and behavioral processes was 0.786 and 0.817, respectively.

##### DB subscale

The DB subscale was used to assess the importance an individual placed on effectively managing depression and determine the importance of taking action for an individual. It comprised 12 items and two dimensions that included perceived benefits and perceived barriers. Each item was scored from 1 (not important) to 5 (extremely important). The Cronbach's alpha values of the perceived benefits and perceived barriers were 0.690 and 0.700, respectively.

##### SE subscale

The SE subscale consisted of 6 items. Each item was scored from 1 (no confidence at all) to 5 (full of confidence), and the scores reflected the degree of confidence in effectively managing depression. Higher scores reflected higher confidence. The Cronbach's alpha of the subscale was 0.735.

#### Secondary outcome measures

##### Social demographic data recording form (SDDRF)

The SDDRF was used to collect patient demographic information such as age, sex, education, disease diagnosis and treatment-related data, such as cardiac function.

### Statistical method

All statistical analyses were completed using SPSS 18.0. Descriptive statistics were used to report participant demographic data. Chi-square tests was used to compare the differences in means of the outcome variables between the two groups at the 2 time points. An analysis of covariance was used to examine the effects of the intervention on the various indexes of depression management. Repeated measures analysis of variance (ANOVA) were used to evaluate the effects of the intervention and the time-intervention interaction on depression management.

## Results

### Participant characteristics

**Fig. 3:**
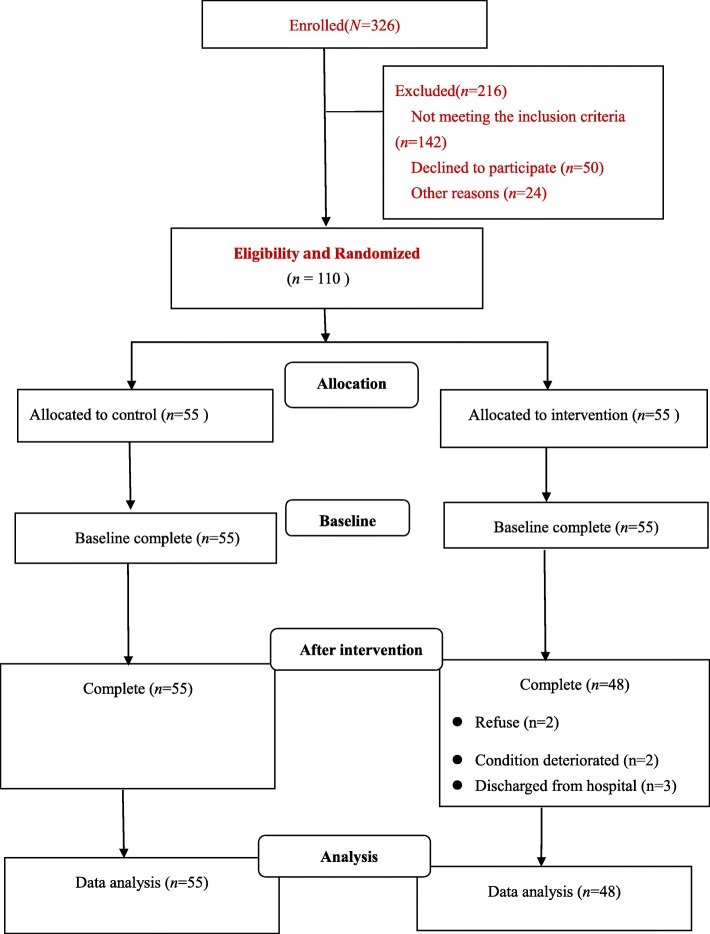
Patient flow diagram

Finally, a total of 103 valid patients were obtained: the control group (*n* = 55) and the intervention group (*n* = 48). The participant flow diagram is shown in Fig. 3. In terms of demographic and clinical characteristics of the samples, the mean age of the participants was 63.3 ± 7.89 years. There were no significant differences in demographic and clinical characteristics between the two groups at baseline (*p* > 0.05) (Table 3). No serious events associated with the intervention occurred throughout the study.

**Table 3:** Demographic and clinical characteristics of the patients

	Intervention Group (***n*** = 48) n (%)	Control Group (***n*** = 55) n (%)	Chi-squared test	
			χ^**2**^	***P***
**Sex**				
Men	30 (62.5)	36 (65.5)	0.100	0.755
Women	18 (37.5)	19 (34.5)		
**Age**				
≤ 60 years	19 (39.6)	17 (30.9)	6.950	0.073
61–65 years	6 (12.5)	18 (32.7)		
66–70 years	14 (10.7)	9 (16.4)		
≥ 71 years	9 (18.8)	11 (20.0)		
**Marital status**				
Married	45 (93.7)	48 (87.3)	1.230	0.268
Widowed or others	3 (6.3)	7 (12.7)		
**Education**				
Primary school	13 (27.1)	14 (25.5)	0.400	0.940
Junior middle school	13 (27.1)	18 (32.7)		
High school or Secondary specialized school	15 (31.3)	16 (29.1)		
College and above	7 (14.6)	7 (12.7)		
**Occupation**				
Workers	24 (50.0)	31 (56.4)	1.240	0.743
Farmers	14 (29.2)	12 (21.8)		
Cadres	8 (16.7)	8 (14.5)		
Others	2 (4.2)	4 (7.3)		
**Type of medical payment**				
Public or medical insurance	34 (70.8)	41 (74.5)	0.180	0.673
Rural cooperative medical care	14 (29.2)	14 (25.5)		
**Cardiac function**				
I	9 (18.8)	9 (16.4)	0.950	0.623
II	19 (39.6)	27 (49.1)		
III	20 (41.7)	19 (34.5)		
**Percutaneous coronary intervention**				
Yes	24 (50.0)	26 (47.3)	0.080	0.782
No	24 (50.0)	29 (52.7)		
**Number of complications**				
One	18 (37.5)	19 (34.5)	0.100	0.952
Two	19 (39.6)	23 (41.8)		
Three	11 (22.9)	13 (23.6)		
**Duration of disease (years)**				
≤ 3	18 (37.5)	14 (25.5)	1.740	0.419
3–5	17 (35.4)	23 (41.8)		
≥ 5	13 (27.1)	18 (32.7)		

### Effect of the intervention on the SOC for CHD depression

#### Inter-group comparisons between the two groups at the two time points (*T* and *T*_*0*_)

A chi-square test was adopted to compare the differences between the two groups at T and T_0_. Table 4 shows that the number of precontemplation patients in the intervention group decreased, while the number of patients in the contemplation, preparation and action stages increased, and these differences between the two groups were significant at T_0_ (*p* = 0.000 < 0.01).

**Table 4:** Comparisons of the number of patients in the stages of change between the two groups at two time points

Stages of change	Intervention group (***n*** = 48) n (%)	Control group (***n*** = 55) n (%)	Chi-squared test	
			χ^**2**^	***P***
***T***				
Precontemplation	30 (62.5)	41 (74.5)	2.551	0.466
Contemplation	16 (33.3)	13 (23.6)		
Preparation	1 (2.1)	1 (1.82)		
Action	1 (2.1)	0 (0.0)		
Maintenance	0 (0.0)	0 (0.0)		
***T***_***0***_				
Precontemplation	15 (31.2)	38 (69.1)	22.198	0.000**
Contemplation	19 (39.6)	16 (29.1)		
Preparation	12 (25.0)	0 (0.0)		
Action	2 (4.2)	1 (1.8)		
Maintenance	0 (0.0)	0 (0.0)		

**p < 0.01; *T* at the baseline, *T*_*0*_ after intervention

#### Intra-group comparison at the two time points for stage changes in the intervention group

A chi-square test was adopted to compare the differences in the number of patients in each stage at the two time points (*T* and *T*_*0*_) in the intervention group. There was a significant difference in the number of patients in each stage at the two time points (*F* = 14.898, *p* = 0.002 < 0.01).

### Effect of the intervention on the various indexes of depression management

#### Comparison of the DB, POC, and SE scores for depression in the CHD patients between two groups after intervention

An analysis of covariance was adopted to examine the effects of the intervention on the DB, POC, SE and depression scores. The regression coefficient test results of perceived benefits, perceived barriers, cognitive level, behavioral level, self-efficacy and depression (*F* = 2.237, *P* = 0.138; *F* = 0.014, *P* = 0.905; *F* = 0.892, *P* = 0.347; *F* = 0.254, *P* = 0.615; *F* = 0.106, *P* = 0.745;*F* = 0.122,*p* = 0.728) meet the basic assumption. Table 5 shows that the scores of the indexes between the two groups were all statistically significantly different after the intervention when excluding the influence of each index score before intervention (*p* < 0.01).

**Table 5:** Comparison of two groups of patients on decisional balance, the process of change, self-efficacy and depression after intervention

Items	Analysis of covariance				Adjusted mean (95%CI)	
	Intervention (***n*** = 48) ($\overline{\mathrm{X}}$ ± s)	Control (***n*** = 55) ($\overline{\mathrm{X}}$ ± s)	F (df)	***P***	Intervention (***n*** = 48)	Control (***n*** = 55)
**Decisional balance**						
Perceived benefits	10.19 ± 2.10	8.73 ± 1.13	33.641 (1.000)	0.000**	10.20 (9.83, 10.57)	8.71 (8.37, 9.06)
Perceived barriers	6.58 ± 1.30	7.93 ± 1.12	18.995 (1.000)	0.000**	6.75 (6.42,7.09)	7.78 (7.47, 8.09)
**Process of change**						
Cognitive level	33.94 ± 5.57	29.35 ± 3.09	67.769 (1.000)	0.000**	34.52 (33.53, 35.51)	28.84 (27.91, 29.76)
Behavioral level	34.44 ± 5.01	32.78 ± 2.87	42.466 (1.000)	0.000**	34.47 (35.37, 37.56)	31.01 (30.00, 32.01)
**Self-efficacy**	12.06 ± 2.40	10.47 ± 1.57	15.443 (1.000)	0.000**	12.00 (12.46, 12.54)	10.53 (10.02, 11.03)
**Depression**	18.46 ± 3.36	20.85 ± 2.28	25.515 (1.000)	0.000**	18.30 (17.53,19.07)	20.99 (20.28,21.71)

**p < 0.01

ANOVA showed that the main effect of the intervention on each dimension of DB, various dimensions of POC and SE showed statistical significance at the two time points except depression (*p* < 0.05). The scores of perceived benefits, perceived barriers, cognitive level, behavioral level, and SE varied with time and showed statistical significance in the main effect of time (*p* < 0.05). There was an interaction between the time factor (*T* and *T*_*0*_) and intervention for each index with the exception of perceived barriers (*p* < 0.05) (Table 6).

**Table 6:** Analysis of the effect of time and intervention on perceived benefits, the process of change and self-efficacy

Items	Intervention main effect		Time main effect		Interaction	
	F (df)	***p***	F (df)	***p***	F (df)	***p***
**Decisional balance**						
Perceived benefits	5.956 (1.000)	0.016*	42.819 (1.000)	0.000**	28.924 (1.000)	0.000**
Perceived barriers	29.134 (1.000)	0.000**	5.702 (1.000)	0.019*	1.327 (1.000)	0.252
**Process of change**						
Cognitive level	4.659 (1.000)	0.033*	132.663 (1.000)	0.000**	74.881 (1.000)	0.000**
Behavioral level	12.517 (1.000)	0.001**	164.765 (1.000)	0.000**	129.878 (1.000)	0.000**
**Self-efficacy**	10.535 (1.000)	0.002**	106.989 (1.000)	0.000**	10.923 (1.000)	0.001**
**Dpression**	0.170 (1.000)	0.681	74.730 (1.000)	0.000**	14.199 (1.000)	0.000**

*p < 0.05, **p < 0.01

### Levels of depression throughout the study

The depression scores decreased more in the intervention group than in the control group. In addition, there were statistically significant differences in the depression scores at different time points in the intervention group (*F* = 17.814, *p* = 0.000 < 0.01).

## Discussion

The results showed after the intervention there were significantly more positive changes in the SOC, POC, DB (more perceived benefits, fewer perceived barriers), SE (higher) and depression (lower) measures in the intervention group than in the control group. These positive effects were consistent with previous studies about depression [31] and stress management [20, 32]. These findings were in line with the results of similar studies, suggesting this model intervention was effective [33–35].

### Effect of the intervention on the SOC for depression in patients with CHD

The majority of patients were in the precontemplation stage (intervention group = 62.5% (30/48); control group = 74.5% (41/55)) at baseline. The number of patients in the intervention group in the precontemplation, contemplation, action stages increased after the intervention, and in the control group, there were almost no improvements. This positive effect was consistent with previous studies in depression [31] and stress management [32], which found that the change stages moved forward after the intervention.

The TTM points out that whether an individual’s behavior change can be transferred from the previous stage to the next stage depends on the change process at each stage, including at the cognitive and behavioral levels [36]. Patients in the precontemplation stage may initially be uninformed or insufficiently aware of the consequences of their behaviors, and some people do not view the behavior as a problem [37]. At the beginning of the study, we found through MI that most of the patients with CHD did not understand what was depression, especially patients with a relatively low educational level who may have never heard of depression. Half of the patients did not know of the harmful consequences of depression on CHD. In addition, under the influence of Chinese Confucianism, some people were unwilling to admit that they had depression. Therefore, in view of these characteristics, the TTM-based intervention focused on providing relevant knowledge about depression and CHD, enabling patients to understand the importance and benefits of prevention and management of depression on physical and mental health and trying to adopt effective methods. For patients in the stages of preparation, action, and maintenance, MI focused on strengthening patient confidence in behavior change to promote patient progress though the stages of change.

### Effect of the intervention on the DB of depression in patients with CHD

The results showed that the patients in the intervention group demonstrated more perceived benefits and fewer perceived barriers than the control group after the intervention. This finding is in line with the results of similar studies, as this model intervention was effective [38].

DB reflects the individual’s relative weighing of the pros and cons of changing [39]. There are two dimensions: the perceived benefits (the benefits and reasons for the behavioral change) and the perceived barriers (the negative aspects and obstacles to the behavioral change) [19]. During the intervention, the patients often underestimated the benefits of preventing and managing depression. Therefore, in the current study, the researchers incorporated each patient’s characteristics into the intervention to help patients recognize the direct and potential benefits of depression prevention and management, emphasize the most relevant benefits for the patients, and make the patients identify possible barriers, thereby providing the available solutions. In the intervention manual, we provided detailed lists of the benefits of taking effective methods to prevent and manage depression, such as ‘I will feel more healthy’, ‘my sleep will be improved’, ‘I will be more attractive’, ‘the pain will be eased’, etc. In addition, the manual for patients with possible barriers to the process of behavior change also provides the corresponding countermeasures, as a way to “counter” the barrier; for example, a patient may think “depression is my family’s inheritance so I can do nothing for it”, and the counter would suggest “if I’m truly a depressed person at high risk, I should work harder to prevent and manage it”.

### Effect of the intervention on the POC of depression in patients with CHD

This study showed that the increases in the cognitive level and behavioral level in the intervention group was significantly higher than those in the control group. The results were similar to the results of the study by Fallon et al. in which people reported greater use of the cognitive and behavioral level of change [40]. The early stages of SOC (precontemplation, contemplation, and preparation) mainly reflect changes at the cognitive level, which increases the willingness of patients to engage in the behavioral change, the later stages (preparation, action, and maintenance) indicate changes at the behavioral level and reflect the aim to undertake observable and maintainable efforts [41], and the transition from the cognitive level to behavioral level indicates the progress across the SOC [42]. In this study, we designed the corresponding intervention strategies according to the influencing factors at all levels of the change process and adopted different psychological intervention measures for patients at different stages according to the relationship between the SOC and POC. The intervention aimed at the most critical problem of behavior change for the patients in each stage, and the most direct approaches were adopted to achieve the maximum intervention effect. Thus, the goal of transforming patients into the next stage of behavior change in the shortest time can be achieved.

### Effect of the intervention on SE of depression in patients with CHD

SE is defined as how well one can execute courses of action required to deal with prospective situations [43]. SE can strongly affect behavioral change [44]. The people with high self-efficacy will make enough effort, which will lead to successful results if executed well, while those with low self-efficacy may stop working hard and fail early [45]. This study demonstrated that SE in the intervention group increased more than that in the control group after the intervention. These results were consistent with those of Zhu et al. [35], who found that the ESMI group demonstrated a higher exercise SE than the control group. Behavior change is an important outcome variable. The change in cognitive and behavioral changes in patients is an effective evaluation index of this study. This study was aimed at individual behavior change so that each patient can easily see his or her progress, then he or she can establish enough self-confidence and persist in the behavioral change to alleviate depression.

This study showed that the depression scores in the CHD patients in the control group decreased to a certain extent. The depression of the patients may be related to the disease itself [28], and if the disease was controlled after treatment, the depression level consequently decreased. However, the depression in the intervention group decreased more than that in the control group due to the additional psychological intervention.

To summarize, these findings suggest that a TTM-based intervention and face-to-face MI were effective in the management of depression in hospitalized patients with CHD. However, the study had three limitations: the first limitation was no long-term follow-up. Therefore, future studies should pay more attention to long-term follow-up to fully verify the progress of all the theoretical stages of TTM and MI. The second limitation was no blinding of the patients, i.e., it was a single-blind trial. Finally, we did not consider the significance of subclinical symptoms of depression. Considering the subclinical symptoms, studies examining depression should be using more than one measurement tool in the future.

## Conclusion

This study confirms that a psychological TTM-based intervention and MI could produce positive effects on depression in hospitalized patients with CHD. The intervention could raise the cognitive level, behavioral level, perceived benefits, and SE, and reduce perceived barriers. This finding will provide guidance for future patient depression and behavior management.

## Data Availability

Data are available from the authors on reasonable request.

## Abbreviations

CHD: Coronary heart disease
TTM: Transtheoretical model;
MI: Motivational interviewing
SOC: Stages of change
POC: Process of change
DB: Decisional balance
SE: Self-efficacy
SDDRF: Social Demographic Data Recording Form
HRSD: Hamilton Rating Scale for Depression
*T*: Patients on admission (at baseline)
*T*_*0*_: Patients 2 days before discharge and after the intervention
